# Prolonged Disease Course of COVID-19 in a Patient with CTLA-4 Haploinsufficiency

**DOI:** 10.1155/2023/3977739

**Published:** 2023-05-22

**Authors:** T. W. Hoffman, H. L. Leavis, B. M. Smits, L. T. van der Veken, D. A. van Kessel

**Affiliations:** ^1^Department of Pulmonology, St. Antonius Hospital, Koekoekslaan 1, 3435 CM, Nieuwegein, Netherlands; ^2^Department of Rheumatology and Clinical Immunology, University Medical Center Utrecht, Utrecht University, Heidelberglaan 100, 3584 CX, Utrecht, Netherlands; ^3^Department of Genetics, Division Laboratories, Pharmacy and Biomedical Genetics, University Medical Center Utrecht, Utrecht University, Heidelberglaan 100, 3584 CX, Utrecht, Netherlands; ^4^Department of Pulmonology, Division of Heart and Lungs, University Medical Center Utrecht, Utrecht University, Heidelberglaan 100, 3584 CX, Utrecht, Netherlands

## Abstract

Patients with primary immunodeficiencies are especially vulnerable to developing severe coronavirus disease 2019 (COVID-19) after infection with the severe acute respiratory syndrome coronavirus 2 (SARS-CoV-2). Cytotoxic T lymphocyte antigen-4 (CTLA-4) is an important regulator of immune responses, and patients who suffer from *CTLA4* haploinsufficiency have hyperactivation of effector T cells and infiltration of various organs. Overexpression of *CTLA4* has been associated with a more severe disease course in patients with COVID-19, but there have only been a few reports on the disease course of COVID-19 in patients with *CTLA4* haploinsufficiency. We report on a 33-year-old female with a history of immune thrombocytopenia, autoimmune haemolytic anaemia, granulomatous-lymphocytic interstitial lung disease, and common variable immunodeficiency who developed COVID-19. She was admitted and discharged from the hospital several times in the months thereafter and remained symptomatic and had a positive SARS-CoV-2 PCR for up to 137 days after the first symptoms. No SARS-CoV-2 antibodies were identified in the patients' serum. The disease was finally controlled after repeated infusions of convalescent plasma and treatment of concurrent bacterial and fungal infections. Genetic analysis revealed a likely pathogenic variant in *CTLA4*, and CTLA4 expression on regulatory T-cells was low. This case illustrates that patients with primary immunodeficiencies who have a protracted disease course of COVID-19 could benefit from convalescent plasma therapy.

## 1. Introduction

Patients with primary immunodeficiencies can be especially vulnerable to developing severe coronavirus disease 2019 (COVID-19) after infection with the severe acute respiratory syndrome coronavirus 2 (SARS-CoV-2) [1, 2]. Most patients with immunodeficiencies are able to clear the virus with or without supportive therapies. However, some patients have a prolonged disease course with viral shedding and symptoms persisting for months [3, 4]. The optimal treatment for COVID-19 in patients with primary immunodeficiency remains to be determined, but various case series report on successful treatment with convalescent plasma [5].

Cytotoxic T lymphocyte antigen-4 (CTLA-4) is an important regulator of immune responses, and patients who suffer from *CTLA4* haploinsufficiency have hyperactivation of effector T cells and infiltration of various organs. Furthermore, patients have decreased circulating B-cells but increased auto-reactive B-cells [6]. Clinical phenotypes of *CTLA4* haploinsufficiency include bronchiectasis, granulomatous-lymphocytic interstitial lung disease (GLILD), autoimmune haemolytic anaemia, autoimmune thrombocytopenia, autoimmune neutropenia, enteropathy, type 1 diabetes, autoimmune thyroiditis, arthritis, psoriasis, malignancy, and lymphocytic infiltration of nonlymphoid organs [7].

Overexpression of *CTLA4* has been associated with a more severe disease course in patients with COVID-19 [8, 9], but there have only been a few reports on the disease course of COVID-19 in patients with *CTLA4* haploinsufficiency [1]. Here, we report the case of a patient with *CTLA4* haploinsufficiency who had a prolonged disease course of COVID-19 that was eventually controlled after multiple infusions of convalescent plasma. The patient provided written informed consent for publication of this report.

## 2. Case Presentation

A 33-year-old female presented to the emergency department with COVID-19. Her past medical history noted that she had recurrent respiratory tract infections since puberty. Furthermore, she had immune thrombocytopenia when she was 20 years old, which was treated with prednisone. Nine months thereafter, she was treated with rituximab for a relapse of immune thrombocytopenia and also autoimmune haemolytic anaemia. When she was 22 years old, she had septic arthritis and was diagnosed with panhypogammaglobulinemia. Additional immune status investigation revealed a severely impaired polysaccharide vaccination response [10] (Table 1). She started monthly substitution therapy with intravenous immunoglobulins. The patient was referred for genetic analysis of her immunodeficiency at that time, but this did not reveal any abnormalities.

**Figure 1:**
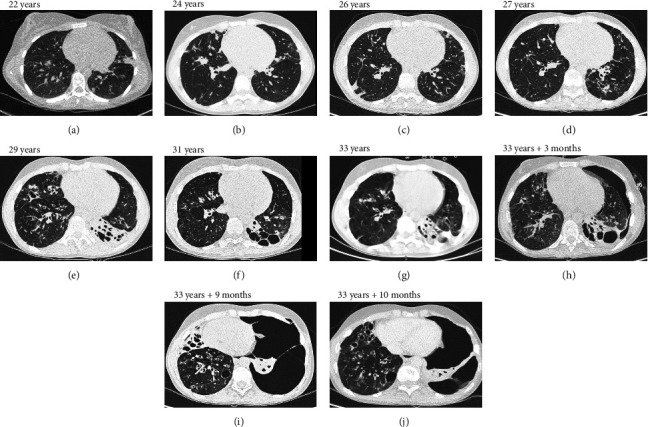
chest computed tomography scan at age 22 years, as well as multiple scans during follow up over the years. The scan at age 33 years was made when the patient presented to the emergency department with COVID-19. (a) Bilateral consolidations that are not well circumscribed, multiple nodules, fissural and pleural irregularities and multiple ground glass areas. Diffuse thickening of bronchial walls. Overall appearance suggestive of granulomatous disease. (b) Disappearance of multiple consolidations, and appearance of multiple new consolidations and ground glass abnormalities. (c) Disappearance of some consolidations in the lower fields, but appearance of new consolidations in the upper fields. (d) Disappearance of some consolidations and appearance of new consolidations and ground glass. (e) New consolidation in the left lower lobe. (f) Impressive progression of bronchiectasis in the left lower lobe, disappearance and appearance of consolidation and ground glass. (g) New consolidations and ground glass, mainly in the lower fields. (h) Pneumothorax on the left side, increase in ground glass and consolidations in both lungs. (i) Increase in pneumothorax on the left side, mediastinal shift to the right, increased bronchiectasis in the middle lobe. (j) Increased air in left upper lobe.

Thereafter, she did not have recurrent respiratory tract infections, but she did show a gradual decline in her lung function, especially the diffusion capacity of the lung for carbon monoxide (DLCO). On chest computed tomography (CT) scans, multiple consolidations and ground glass areas were seen in the lower fields, as well as pleural and fissural irregularities, most consistent with a granulomatous disease (Figure 1(a); further CT-imaging in the course of the patient's life is provided in Figures 1(b)–1(j)). When she was 24 years old, she underwent a lung biopsy through video-assisted thoracoscopic surgery, which did not lead to a classifying diagnosis but only revealed mild reactive changes. Afterwards, she had recurrent respiratory tract infections, including sinusitis, for which she underwent functional endoscopic sinus surgery at age 25. At age 27, she was treated with prednisone maintenance therapy, initially at 15 mg/day and later at 5 mg/day. She had *pneumocystis jirovecii* pneumonia shortly after initiation of prednisone therapy but recovered after antibiotic treatment. At age 29, she had septic arthritis again and was still having recurrent respiratory tract infections.

During the present episode (spring 2020, during the first wave of the COVID-19 pandemic in The Netherlands), she had a productive cough for seven days and a fever for one day. She had also experienced upper respiratory tract symptoms and a headache. On physical examination, the patient was febrile, with a temperature of 39.2 degrees Celsius and oxygen saturation while breathing ambient air of 91%. A CT-scan of the chest was performed, which revealed pre-existent bronchiectasis with new consolidations and ground glass abnormalities in the lower fields (Figure 1(f)). A pharyngeal swab analysed by the SARS-CoV-2 polymerase chain reaction came back positive.

The patient was admitted to the hospital with COVID-19 and was started on supplemental oxygen (2 L/minute), cefuroxime, ciprofloxacin, and chloroquine. In the next few days, her condition improved, and she could be weaned off from supplemental oxygen. *H. influenzae*, resistant to cefuroxime, was grown from a sputum culture, and antibiotics were switched to oral cotrimoxazole. The patient could be discharged home five days after admission.

Nine days after discharge, she presented to the emergency department again with persistent fever and a left-sided pneumothorax, with a maximum thickness of 22 mm. The SARS-CoV-2 PCR on a pharyngeal swab was positive. The patient was readmitted and treated for a presumed bacterial pneumonia with cefotaxime for seven days. No intervention was performed for the pneumothorax. On a bronchoscopy for bronchial washings no bacteria were cultured. A chest CT scan showed persistent ground glass abnormalities in both lungs, in addition to a small left-sided pneumothorax and a right-sided consolidation (Figure 1(g)). After admission, the patient showed slow clinical improvement, and the pneumothorax regressed. She was discharged fifteen days after admission.

Twelve days after discharge from the second hospitalization, the patient presented to the emergency department again with a persistent fever. A chest X-ray showed a right-sided consolidation. A pharyngeal swab sent for SARS-CoV-2 PCR came back positive. Because the patient had relatively mild symptoms at that time, she was treated as an out-patient with oral amoxicillin/clavulanic acid. However, fevers did not subside, and the patient complained of an increased cough. From sputum, *S. aureus* was cultured and the patient was treated with flucloxacillin, with initial improvement of symptoms.

However, two weeks later (eleven weeks after the initial emergency department presentation), the patient was admitted for the third time with an increased cough, recurring fever, and acute-onset dyspnoea. A chest X-ray revealed a left-sided pneumothorax that had enlarged to 44 mm (from <5 mm) and a persistent right-sided consolidation. A chest drain was placed under local anaesthesia, and cefuroxime was started. Due to hemodynamic instability, the patient was admitted to the intensive care for inotropic support.

The SARS-CoV-2 PCR on a pharyngeal swab was positive again, as was the PCR for rhinovirus. *A. fumigatus* was isolated from a previously submitted sputum culture. SARS-CoV-2 antibodies in the patients' serum were found to be very low. The patient was started on voriconazole because of concern for pulmonary aspergillosis, even though she did not fulfill criteria for probable or possible invasive fungal disease. Furthermore, she received two infusions with donor convalescent plasma at a two-week interval. The patient slowly improved. After transfer to the pulmonology ward, two more infusions of convalescent plasma were repeatedly administered. Voriconazole was discontinued. Due to the recurrence of the pneumothorax, a bullectomy and pleurectomy were performed during the mini thoracotomy. Two months after initial admission, she could be discharged home. At that time the fever had disappeared and she was feeling better.

Three repetitive weakly pharyngeal swabs remained negative for SARS-CoV-2 PCR testing. The patient reported a decreased cough and dyspnoea and did not have any more fevers. In the following year, the patient was admitted four more times: once for haemoptysis which was related to bleeding from pathologic bronchial arteries and for which bronchial artery embolization was performed two times for bacterial pneumonia and a fourth time for a recurrent pneumothorax, for which a chest drain was placed. Multiple pharyngeal swabs and sputum samples tested for SARS-CoV-2 PCR were negative.

However, despite being vaccinated with the Pfizer/BioNTech SARS-CoV-2 vaccine twice (10 and 11 months after the initial presentation with COVID-19), the patient did not have detectable anti-SARS-CoV-2 antibodies at 10 months after the initial presentation with COVID-19 (Table 2).

Because of the protracted disease course of COVID-19, the patient was referred for lymphocyte subset analysis. This revealed 0 B cells, low CD4+, CD8+, and naive T cells (Table 3). Furthermore, next-generation sequencing was performed, with filtering of the results for 426 genes associated with primary immunodeficiencies (PID00v20.2; Utrecht University Medical Center). This revealed an in-frame deletion-insertion variant in the *CTLA4* gene (c.231_c297delinsA p.(Gln80_Ser101del)) classified as likely pathogenic. This variant has not been found in ∼140000 healthy controls and was not previously described in the ClinVar Database (https://www.ncbi.nlm.nih.gov/clinvar/; now submitted under ClinVar accession ID VCV001801487.1) or Human Gene Mutation Database (https://www.hgmd.cf.ac.uk/ac/index.php). No pathogenic or likely pathogenic variants were found in the other genes in the gene panel associated with primary immunodeficiencies.

**Figure 2:**
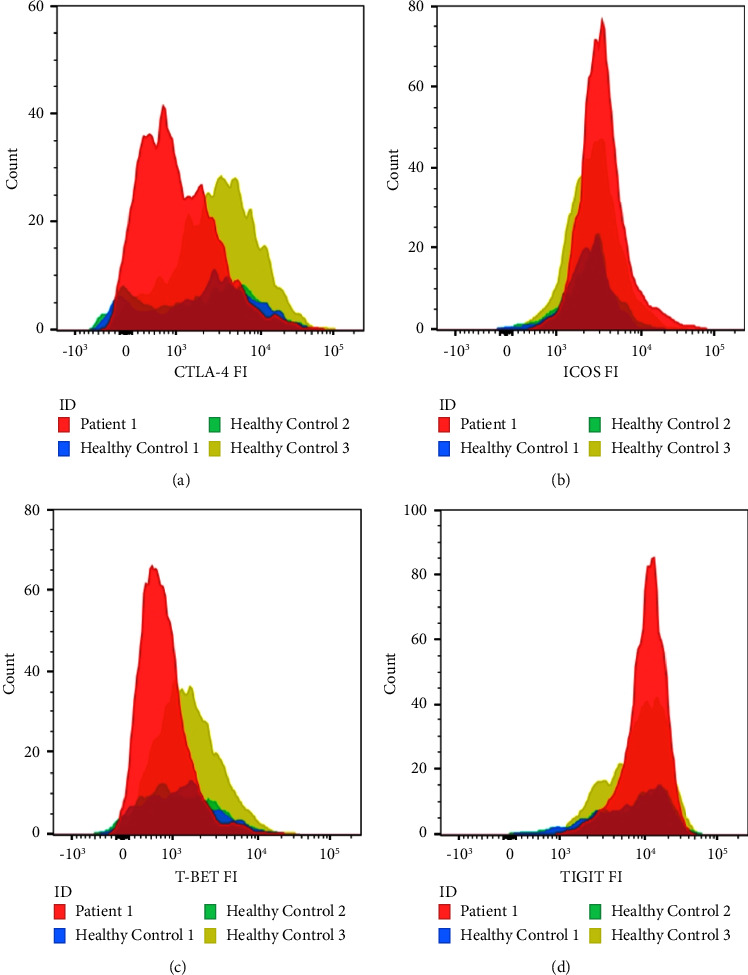
(a) CTLA4 expression in CD25+ FOXP3+ T-cells within CD4+ population in our patient (in red), as well as healthy controls (in blue, green, and yellow). (b) ICOS expression in CD25+ FOXP3+ T-cells within CD4+ population in our patient and healthy controls. (c) Expression in CD25+ FOXP3+ T-cells within CD4+ population in our patient and healthy controls. (d) Expression in CD25+ FOXP3+ T-cells within CD4+ population in our patient and healthy controls.

Flow cytometry was performed on peripheral blood mononuclear cells as previously described [11]. Of all CD4+-cells, 7.2% were regulatory T-cells. The mean fluorescence intensity of CTLA-4 on our patients' regulatory T-cells was 1057, which was lower than the 95% confidence interval calculated for healthy controls (2038–3191, Figure 2).

## 3. Discussion

This case illustrates that patients with primary immunodeficiencies can have a protracted disease course of COVID-19. This has been the subject of several previous reports [1, 3, 12], but the SARS-CoV-2 PCR positivity of 137 days in our patient is especially long in comparison. Two previously reported patients with CTLA4-haploinsufficiency who developed COVID-19 seemed to have a shorter disease course, although the duration of SARS-CoV-2 PCR positivity for these patients was not reported [1].

That some patients with immunodeficiency have such a protracted disease course, and other do not likely depends on the precise immune defect. It is known that patients with primary immunodeficiencies are at risk for severe, recurrent, and sustained viral infections with viruses other than SARS-CoV-2 [13].

The optimal treatment for immunocompromised patients with COVID-19 remains to be determined. The use of convalescent plasma does seem beneficial in immunocompromised patients without antibodies to SARS-CoV-2, but evidence for any specific type of immunodeficiency is still anecdotal [5, 14]. At what time in the course of the disease convalescent plasma could be given best, as well as the optimal dose and dosing interval, is still unclear. It is also not clear how long convalescent plasma should be given in cases with a protracted disease course who have relatively mild symptoms. The strategy that was employed in this patient, where regular SARS-CoV-2 PCR tests were done and the convalescent plasma was given two more times after the last positive SARS-CoV-2 PCR and clinical improvement of the patient, seems reasonable. Monoclonal antibodies against SARS-CoV-2 have been developed and have been used in immunocompromised patients as well [15]. However, these could not be used in this case, as they were not available at the time our patient was treated with convalescent plasma. In addition, viral escape from monoclonal antibodies over time has proven problematic, and high-titre polyclonal convalescent plasma seems promising despite viral shift (as currently tested in the convalescent plasma domain within the REMAP-CAP trial [16]). Of note, our patient probably received few antibodies against SARS-CoV-2 via her monthly intravenous immunoglobulin substitution therapy during the period in which she received convalescent plasma, as the overall seroprevalence of antibodies against SARS-CoV-2 in that period was only around 5% [17].

In retrospect, the overall clinical picture of our patient is consistent with CTLA4-haploinsufficiency. Immune thrombocytopenia and autoimmune haemolytic anaemia together can be seen in the context of Evans' syndrome, which can be seen in patients with CTLA4-haploinsufficiency [18]. GLILD and the patient's immunodeficiency are also consistent with CTLA4-haploinsufficiency, which is usually characterized by antibody deficiency and low levels of naive T-cells [7]. The diagnosis of CTLA4-haploinsufficiency was made relatively late for our patient. The patient had been referred for genetic analysis of her immunodeficiency more than 10 years prior to developing COVID-19, which did not reveal any abnormalities. However, the gene panel at that time did not include *CTLA4*. Renewed genetic screening was prompted by the protracted disease course of COVID-19.

In conclusion, we report on a patient with a history of immune thrombocytopenia, autoimmune haemolytic anaemia, GLILD, and CVID, who developed COVID-19. She remained symptomatic and had a positive SARS-CoV-2 PCR for up to 137 days after the first symptoms. However, the disease was finally controlled after repeated infusions of convalescent plasma and treatment of concurrent bacterial and fungal infections. Genetic analysis revealed a likely pathogenic variant in *CTLA4*, CTLA4-expression on regulatory T-cells was confirmed to be low (as reported in CTLA4-haploinsufficiency by Schubert et al. [19]), and the entire clinical picture is consistent with CTLA4-haploinsufficiency.

## Figures and Tables

**Table 1:** Immune status investigations when the patient was 22 years old, prior to starting intravenous immunoglobulin substitution therapy.

	Patient's result	Normal range
IgA (g/L)	0.24	0.70–4.00
IgM (g/L)	<0.30	0.40–2.30
IgG (g/L)	2.74	7.00–16.00
IgG1 (g/L)	2.60	4.90–11.40
IgG2 (g/L)	0.20	1.50–6.40
IgG3 (g/L)	0.50	0.20–1.10
IgG4 (g/L)	0.02	0.08–1.40
Complement AP (%)	115	30–115
Complement CP (%)	128	70–130
Complement MP (%)	145	10–125
Response to pneumococcal polysaccharide vaccination	Severely impaired	Normal
Toxoplasma IgG (IU/mL)	<2	>7.2
Diphtheria antibodies (IU/mL)	0.03	>0.01
Rubella IgG (IU/mL)	11	>10
Tetanus antibodies (IU/mL)	0.38	>0.01

Interpretation of the response to pneumococcal vaccination was based on the 2015 AAAAI/ACAAI criteria. [10]. AP = alternative pathway; CP = classical pathway; MP = lectin pathway.

**Table 2:** SARS-CoV-2 PCR test results, anti-SARS-CoV-2 antibody levels, and convalescent plasma infusions.

Days after start of first symptoms of COVID-19	Protective antibody level	Antibody level (A/CO)	SARS-CoV-2 PCR	Ct-value	Sample
7			Positive	24.6	Oropharynx
20			Positive	30.8	Oropharynx
25	Negative	0.020			
32			Negative	NA	Bronchial washing
33			Positive	NA	Nasopharynx
49			Positive	NA	Nasopharynx
61			Positive	29.7	Sputum
83			Positive	23.8	Oropharynx
88	Negative	0.024			
89		First transfusion of convalescent plasma			
89	Positive	4.830			
91			Positive	NA	Oropharynx
94			Positive	32.4	Oropharynx
96	Negative	0.527			
98	Negative	0.381			
101			Positive	28.1	Oropharynx
103		Second transfusion of convalescent plasma			
110	Positive	9.083	Positive	27.6	Oropharynx
116	Positive	10.203	Positive	38.9	Oropharynx
119		Third transfusion of convalescent plasma			
123	Positive	7.437	Negative	NA	Oropharynx
126			Positive	28.9	Oropharynx
130			Negative	NA	Oropharynx
131	Positive	4.523			
133		Fourth transfusion of convalescent plasma			
136			Positive	NA	Oropharynx
137	Positive	13.935	Positive	36.8	Oropharynx
143	Positive	12.567			
144			Negative	NA	Oropharynx
146			Negative	NA	Oropharynx
146		Fifth transfusion of convalescent plasma			
153			Negative	NA	Oropharynx
165	Positive	>14	Negative	NA	Oropharynx
165		Sixth transfusion of convalescent plasma			
181	Positive	>14	Negative	NA	Oropharynx
196			Negative	NA	Oropharynx
207	Positive	10.602			
236	Positive	4.050	Negative	NA	Oropharynx
275			Negative	NA	Oropharynx
291		First SARS-CoV-2 vaccine			
300	Negative	<3.80 (IgG (AU/mL))			
319		Second SARS-CoV-2 vaccine			
384	Negative	<1.85 (IgG (AU/mL))			
414			Negative	NA	Oropharynx
417	Negative	<1.85 (IgG (AU/mL))			
553			Negative	NA	Sputum

Reference numbers for antibody levels are >1.1 A/CO and >13 AU/mL. Ct = cycle threshold. NA = not available.

**Table 3:** Lymphocyte subset analysis performed when the patient was 34 years old and had recovered from COVID-19.

	Patient's result	Normal range
T cells (/*μ*L)	251	700–1900
CD4+ (/*μ*L)	117	560–1067
CD8+ (/*μ*L)	144	216–499
CD4/CD8-ratio	0.8	1.4–4.0
Activated T cells (% within CD4)	1.0	0.3–1.0
Naive T cells (% within CD4)	15.5	49.4–71.9
Effector T cells (% within CD4)	84.5	27.3–49.8
Activated T cells (% within CD8)	0.6	0.9–4.2
Naive T cells (% within CD8)	13.1	48.6–87.5
Effector T cells (% within CD8)	86.9	11.7–42.9
B cells (/*μ*L)	0	114–436
NK cells (/*μ*L)	36	100–400

## Data Availability

The data used in this study are made available upon reasonable request to the corresponding author.
